# Treatment of Listeriosis in First Trimester of Pregnancy 

**DOI:** 10.3201/eid1905.121397

**Published:** 2013-05

**Authors:** Brian T. Chan, Elizabeth Hohmann, Miriam Baron Barshak, Read Pukkila-Worley

**Affiliations:** Massachusetts General Hospital, Boston, Massachusetts, USA (B. T. Chan, E. Hohmann, M. B. Barshak, R. Pukkila-Worley);; Brigham and Women’s Hospital, Boston (B.T. Chan)

**Keywords:** Listeriosis, pregnancy, antibiotics, antimicrobial drugs, Listeria monocytogenes, bacteria, zoonosis

**To the Editor:** Foodborne infections with *Listeria monocytogenes* continue to be dangerous and disruptive. A 2011 outbreak in the United States, linked to cantaloupes, affected 147 persons; 33 persons died, and 1 pregnant woman experienced a miscarriage (*1*). Moreover, the incidence of listeriosis has been rising in several European countries (*2*). Compared with the general population, pregnant women are at markedly increased risk of acquiring listeriosis (*3*). Women who are infected with *L. monocytogenes* in the third trimester of pregnancy are typically treated with antimicrobial drugs until the child’s delivery (*3*). However, the optimal treatment regimen for listeriosis early in pregnancy is unknown.

We cared for a 28-year-old, previously healthy woman who sought treatment at 12 weeks’ gestational age with fever, headache, and neck stiffness; blood cultures were positive for *L. monocytogenes*. Lumbar puncture on admission to our hospital in Boston, Massachusetts, in December 2011, revealed clear fluid and an opening pressure of 15 mm Hg; 1 leukocyte was observed per high-powered field, and cultures of the cerebrospinal fluid were sterile. Pelvic ultrasound showed no abnormalities of the fetus, gestational sac, or uterus.

We treated the patient’s condition with intravenous ampicillin for 2 weeks, 2 g every 4 hours, and gentamicin, 100 mg every 8 hours, followed by ampicillin alone for 2 weeks. Shortly after the antimicrobial drugs were initiated, the patient defervesced and her blood cultures cleared. Her hospital course was complicated by spinal headache and transient acetaminophen-induced liver injury, but she was eventually discharged to her home in good condition. Blood cultures taken after discontinuation of antimicrobial agents were sterile, and the remainder of her pregnancy was unremarkable.

She ultimately gave birth to a healthy 2,405-g boy with Apgar scores of 4 and 7 (at 1 and 5 min, respectively) at 35.1 weeks’ gestation by spontaneous vaginal delivery. Pathologic examination of the placenta showed no evidence of chorioamnionitis, villitis, or parenchymal abscesses, and placental cultures were sterile. The patient and her child are currently doing well without obvious sequelae of infection.

Listeriosis in early pregnancy presents a unique challenge for the infectious diseases clinician. Up to 30% of *L. monocytogenes* infections in pregnancy result in stillbirth, miscarriage, or preterm labor, and approximately two thirds of surviving neonates are infected (*4*). *L. monocytogenes* uses 2 surface proteins, InlA and InlB, to invade host cells, including the placenta (*5*). Once established within the placenta, *L. monocytogenes* forms microabscesses, which can lead to recurrence of infection (*6*). A recent study in which researchers used a guinea pig model suggests that eradication of microabscesses from the placenta may be critical to achieving the cure of the mother and the prevention of fetal illness and death (*7*).

What, then, is the optimal treatment strategy to cure the mother and sterilize the placenta? In a large case series of pregnant women with listeriosis, most patients were given a β-lactam antimicrobial drug, with or without gentamicin (*6*). However, most women in this case series were in their third trimester of pregnancy and received treatment until delivery. In women who are infected in the first or second trimester, continuing intravenous antimicrobial drugs until delivery is impractical, and the efficacy of oral antimicrobial agents in preventing recurrence of infection is unknown.

Our case demonstrates that 4 weeks of intravenous therapy can sterilize the placenta and enable good maternal and fetal outcomes in a woman infected with listeriosis in the first trimester. We also identified 13 case reports of women in whom listeriosis developed in the first or second trimester of pregnancy (Technical Appendix). Among these 13 case-patients, 8 instances occurred in which both mother and neonate survived without sequelae; all 8 patients had received ampicillin/penicillin with or without gentamicin.

The role of gentamicin in treatment of listeriosis in pregnancy is controversial. The combination of ampicillin and gentamicin has been thought to be synergistic, although in vivo evidence of clinical benefit, compared to that of treatment with ampicillin alone, is lacking (*3*,*6*). A particular concern in pregnancy is gentamicin’s poor penetration into the intracellular space, where *L. monocytogenes* is likely to reside, in the placenta (*8*). Furthermore, some concern exists that gentamicin use in pregnancy could cause fetal ototoxicity, although few such cases have been reported, and several small cohort studies have not shown this association (*9*,*10*). Our patient’s child had a normal result when standard audiology testing was performed several days after delivery.

Infectious diseases clinicians will likely see patients with listeriosis in early pregnancy, given the increasing incidence of this infection in many countries and the ongoing threat of food-borne outbreaks. The collected experience from the cases reported here may be useful, particularly given the absence of high quality clinical data that support treatment recommendations for this population. Intravenous ampicillin, with or without gentamicin, effectively sterilizes the placenta and prevents maternal and fetal illness and death in cases of listeriosis in early pregnancy.

Technical Appendix Table that describes the treatment of listeriosis in 13 pregnant women in the first and second trimesters of pregnancy, 1961–2002
