# Supplementary material for: Treatment of Listeriosis in First Trimester of Pregnancy
Source: Emerg Infect Dis. 2013 May;19(5):839–41. doi: 10.3201/eid1905.121397 (PMC3647505; doi:10.3201/eid1905.121397)
Supplement: Technical Appendix — Table that describes the treatment of listeriosis in 13 pregnant women in the first and second trimesters of pregnancy, 1961–2002 [file 12-1397-Techapp-s1.pdf]

Article DOI: http://dx.doi.org/10.3201/eid1905.13997

Treatment of Listeriosis in First Trimester of Pregnancy

Technical Appendix

Technical Appendix Table. Studies describing treatment of listeriosis in pregnant women in first and second trimesters, 1961–2002*

Patient characteristics/ GA of fetus	Treatment	Outcome	Reference
Bacteremic/6 mo	OXT × 1 wk	Preterm delivery; neonate with *Listeria* meningitis	Hood, 1961
Bacteremic/26 wk	AMP × 2 wk	Uncomplicated delivery (induced, 41.5 wk); neonate unaffected	Fleming et al., 1985
Bacteremic/20 wk	PEN × 9 d	Uncomplicated term delivery; neonate unaffected	Zervoudakis and Cederqvist, 1977
Bacteremic/19 wk	AMP × 3 wk	Uncomplicated term delivery; neonate unaffected	Hume, 1976
Bacteremic/18 wk	AMP × 2 wk; then PO AMX	Uncomplicated delivery at 39 wk; neonate unaffected	Benshushan et al., 2002
Bacteremic/18 wk	AMP × 7 d, GEN × 2 d; then PO AMX	Diagnosed postpartum; preterm delivery with fetal demise; mother recovered	Mylonakis et al., 2002
Bacteremic/15 wk	AMP × 3 wk	Uncomplicated delivery at 39 wk by C-section; neonate unaffected	Benshushan et al., 2002
Bacteremic/13 wk	AMP. and GEN × 3 wk	Uncomplicated delivery at 36 wk; neonate unaffected	Fuchs et al., 1994
Bacteremic/13 wk	AMP × 10 d, GEN × 6 d, then TMP/SXT × 10 d	Uncomplicated term delivery; neonate unaffected	Cruikshank and Warenski,1989
Bacteremic/12 wk	AMP and GEN x 2 wk, then AMP × 2 wk	Uncomplicated delivery at 35 wk; neonate unaffected	This study
+ Uterine culture/27 wk	AMP and GEN × 2 d, then ERY × 1 wk	Preterm delivery; neonate unaffected	Mylonakis et al., 2002
+ Uterine culture/18 wk	CLI and GEN	Sought treatment with contractions and fever; spontaneous abortion resulted; acute chorioamnionitis noted	Benshushan et al., 2002
+ Uterine culture/17 wk	AMP and GEN × 2 d, then PO AMP × 14 d	Sought treatment with spontaneous abortion and fetal demise; mother recovered	Mylonakis et al., 2002
+ D&C culture/17 wk	PEN and CVA, followed by AMP, CLI, and GEN	Sought treatment with fever and missed abortion; D&C performed	Benshushan et al., 2002

*GA, gestational age; OXT, oxytetracycline; AMP, ampicillin; C-section, cesarean section; PEN, penicillin; AMX, amoxicillin; PO, per os (by mouth); GEN, gentamicin; TMP/SXT, trimethoprim/sulfamethoxazole; ERY, erythromycin; CLI, clindamycin; D&C, dilatation and curettage; CVA, clavulanic acid.

References

Benshushan A, Tsafrir A, Arbel R, Rahav G, Ariel I, Rojansky N. *Listeria* infection during pregnancy: a 10 year experience. Isr Med Assoc J. 2002;4:776–80. PubMed

Cruikshank DP, Warenski JC. First-trimester maternal *Listeria monocytogenes* sepsis and chorioamnionitis with normal neonatal outcome. Obstet Gynecol. 1989;73:469–71. PubMed

Fleming AD, Ehrlich DW, Miller NA, Monif GR. Successful treatment of maternal septicemia due to *Listeria monocytogenes* at 26 weeks' gestation. Obstet Gynecol. 1985;66(Suppl):52S–3S. PubMed

Fuchs S, Hochner-Celnikier D, Shalev O. First trimester listeriosis with normal fetal outcome. Eur J Clin Microbiol Infect Dis. 1994;13:656–8. PubMed http://dx.doi.org/10.1007/BF01973992

Hood M. Listeriosis as an infection of pregnancy manifested in the newborn. Pediatrics. 1961;27:390–6. PubMed

Hume OS. Maternal *Listeria monocytogenes* septicemia with sparing of the fetus. Obstet Gynecol. 1976;48(Suppl):33S–4S. PubMed

Mylonakis E, Paliou M, Hohmann EL, Calderwood SB, Wing EJ. Listeriosis during pregnancy: a case series and review of 222 cases. Medicine (Baltimore). 2002;81:260–9. PubMed http://dx.doi.org/10.1097/00005792-200207000-00002

Zervoudakis IA, Cederqvist LL. Effect of *Listeria monocytogenes* septicemia during pregnancy on the offspring. Am J Obstet Gynecol. 1977;129:465–7. PubMed
